# Position paper to facilitate patient access to radiopharmaceuticals: considerations for a suitable pharmaceutical regulatory framework

**DOI:** 10.1186/s41181-023-00230-2

**Published:** 2024-01-02

**Authors:** Aruna Korde, Marianne Patt, Svetlana V. Selivanova, Andrew M. Scott, Rolf Hesselmann, Oliver Kiss, Natesan Ramamoorthy, Sergio Todde, Sietske M. Rubow, Luther Gwaza, Serge Lyashchenko, Jan Andersson, Brian Hockley, Ravindra Kaslival, Clemens Decristoforo

**Affiliations:** 1 Division of Physical and Chemical Sciences, Department of Nuclear Sciences and Applications, International Atomic Energy Agency, Vienna, Austria; 2 Section Radiopharmacy, Department of Nuclear Medicine, University Hospital Augsburg, Augsburg, Germany; 3 Canadian Nuclear Laboratories, Chalk River, ON Canada; 4 Faculty of Pharmacy, Universite Laval, Quebec City, QC Canada; 5 Department of Molecular Imaging and Therapy, Austin Health, and Faculty of Medicine, University of Melbourne, Melbourne, Australia; 6 Olivia Newton-John Cancer Research Institute, and School of Cancer Medicine, La Trobe University, Melbourne, Australia; 7 Health Protection Directorate, Radiation Protection Division, Section for Research Facilities and Nuclear Medicine, Federal Office of Public Health, Bern, Switzerland; 8 Department of Targetry, Target Chemistry and Radiopharmacy, Institute for Radipopharmaceutical Cancer Research, Helmholtz-Zentrum Dresden-Rossendorf (HZDR), Dresden, Germany; 9 National Institute of Advanced Studies (NIAS), Bangalore, 560012 India; 10 Department of Medicine and Surgery, University of Milano-Bicocca, Tecnomed Foundation, Via Pergolesi, 33, 20900 Monza, Italy; 11 Faculty of Medicine and Health Sciences, Stellenbosch University, Cape Town, South Africa; 12 Health Products Policy and Standards Department, World Health Organization, Geneva, Switzerland; 13 Department of Radiology, Memorial Sloan Kettering Cancer Center, New York, NY USA; 14 Edmonton Radiopharmaceutical Centre, Alberta Health Services, Edmonton, Canada; 15 Department of Oncology, University of Alberta, Edmonton, Canada; 16 Division of Nuclear Medicine, Department of Radiology, University of Michigan, Ann Arbor, MI USA; 17 Office of New Drug Products, Office of Pharmaceutical Quality, CDER, U.S. Food and Drug Administration, Silver Spring, MD USA; 18 Department of Nuclear Medicine, Medical University Innsbruck, Anichstrasse 35, 6020 Innsbruck, Austria

**Keywords:** Radiopharmaceutical, Regulations, Legislation, Regulatory framework, GMP, Marketing authorisation

## Abstract

**Background:**

Nuclear medicine has made enormous progress in the past decades. However, there are still significant inequalities in patient access among different countries, which could be mitigated by improving access to and availability of radiopharmaceuticals.

**Main body:**

This paper summarises major considerations for a suitable pharmaceutical regulatory framework to facilitate patient access to radiopharmaceuticals. These include the distinct characteristics of radiopharmaceuticals which require dedicated regulations, considering the impact of the variable complexity of radiopharmaceutical preparation, personnel requirements, manufacturing practices and quality assurance, regulatory authority interfaces, communication and training, as well as marketing authorisation procedures to ensure availability of radiopharmaceuticals. Finally, domestic and regional supply to ensure patient access via alternative regulatory pathways, including in-house production of radiopharmaceuticals, is described, and an outlook on regulatory challenges faced by new developments, such as the use of alpha emitters, is provided.

**Conclusions:**

All these considerations are an outcome of a dedicated Technical Meeting organised by the IAEA in 2023 and represent the views and opinions of experts in the field, not those of any regulatory authorities.

## Background

Radiopharmaceuticals (RPs) are a special class of medicinal products. As suggested by their name, RPs have two different components. One is a radioactive isotope (radionuclide), which is responsible for the desired “action” of the RPs—i.e. emitting radiation that can be detected by suitable equipment in the case of diagnostic RPs or causing intended damage and loss of viability to the cells in a pathological environment in the case of therapeutic RPs. The other component of RPs is a suitable “vector” or “carrier molecule” capable of carrying or delivering the radioactive nuclide to the desired location, the targeted organ, tissue or lesion. The vector may be as simple as the radionuclide itself or a complex chemical entity ranging from a small organic molecule to a large peptide, antibody or nanoparticle (Lewis et al. 2019). The independent action of either of the components is not sufficient to exert a diagnostic or therapeutic effect, but when combined, they become a drug (or medicinal product).

Since its establishment in the early 1960s, nuclear medicine has made enormous progress for diagnosis and/or management of a wide variety of pathological conditions, initially with Single Photon Emission Computed Tomography (SPECT) and later with Positron Emission Tomography (PET), and over 40 million nuclear medicine procedures are performed every year worldwide. However, there are still significant inequalities in patient access, with more than 80% of the procedures performed in OECD (Organisation for Economic Co-operation and Development) countries (OECD, Nuclear Energy Agency 2019), and there are entire regions of the world where the preparation and use of RPs are still a prerogative of very few healthcare establishments, with technologies and methodologies often being outdated.

Moreover, the field of nuclear medicine has seen dramatic changes and advances in the last two decades; new diagnostic and therapeutic applications of novel radiopharmaceuticals, which proved to be safe and clinically effective, have been approved and adopted into clinical practice in a number of countries (Bodei et al. 2022; Sgouros et al. 2020; Weber et al. 2020; Hricak et al. 2021). These developments highlight a clear need for ease of access to proven and emerging radiopharmaceuticals to enable appropriate patient treatment and improved clinical outcomes in many other regions of the world. The pace of innovation and related benefit to patients might potentially be much faster if barriers that still hamper the widespread availability of RPs are removed.

Several factors contribute to making the access to RPs challenging. Amongst these, the pharmaceutical regulatory framework and associated guidelines play a very important role in many ways. First, the peculiarity and uniqueness of RPs are often poorly (or not at all) recognised and not adequately considered. This results in an unsustainable burden, especially in regions with a well-structured regulatory framework and where the preparation and use of RPs is quite common. Moreover, rules may significantly vary from country to country, leading to a lack of harmonisation that further impacts the availability of RPs. According to information gathered through the International Atomic Energy Agency (IAEA) Regional Projects and Technical Meetings (TM), in some countries, especially those with resource constraints, regulation of RPs may be very limited and/or formal regulations may not be in place. This results in lack of clarity on the regulations for RPs, such as their registration, import and compounding in hospital radiopharmacies, even though some RP products are routinely used for nuclear medicine procedures worldwide.

Another challenge in regulating radiopharmaceuticals is an overlap of pharmaceutical and radiation safety requirements, which may lead to apparently conflicting demands of infrastructure provisions (e.g. ventilation requirements in formulation areas); such a situation can be exacerbated by pharmaceutical regulators often having limited knowledge of radionuclide production and radiopharmaceutical preparation. Similarly, radiation safety regulators may not necessarily have the required understanding of and experience in pharmaceutical safety aspects and health care outcomes of the use of RPs. The result is that even for countries where both conventional drug and radioactive material use are adequately regulated individually, it is often demanding to consolidate the two for regulatory oversight of RPs.

Sharing harmonised, simple and adequate guidance, along with any available good practices in regulation, would be very beneficial for most of the national stakeholders. The IAEA provides guidance and advice to its member states (MS) on various aspects of the regulation of radioactive materials, including medical radioisotopes and RPs, such as suitable standards for safe handling and transport of radioactive materials and technical guidance documents on the state-of-the-art equipment and methodologies for preparation and quality control of radionuclides and RPs. The IAEA and World Health Organization (WHO) are collaboratively engaged in preparing Good Manufacturing Practice (GMP) guidance documents and monographs for RPs. Various MS have requested to extend this guidance to include a regulatory framework for RPs. Hence, a multidisciplinary TM was held at the IAEA headquarters, Vienna, during 6–10 March 2023, titled”Health/pharmaceutical regulations for radiopharmaceuticals”. The TM, conducted in hybrid mode, brought together 37 experts in radiopharmaceuticals, including regulators, producers, researchers and clinicians from 19 MS, in addition to WHO, IAEA and OECD representatives. The TM captured the current overview of regulatory practices for RPs in participating MS and discussions focused on the regulatory challenges for small-scale RP production (at nuclear medicine centres and radiopharmacies, including those linked to medical cyclotrons, national labs or enterprises, etc.). This requires special considerations based on clinical demands, expertise, infrastructure and other resources, any associated risks and ensuring patient’s ease of timely access to safe and effective radiopharmaceuticals with appropriate or requisite quality.

Some countries would like to have guidance for strengthening or formalising the pharmaceutical regulatory framework for RPs in routine use elsewhere, while many others seek faster access to new and emerging RPs to deliver timely benefits to patients in need. While recognizing the need for the former (already partly addressed by other IAEA events and documents), the focus of the TM was directed to enabling and ensuring early access to emerging RPs (especially cyclotron-produced RPs for PET/CT imaging and RPs for targeted therapy) as well as”first-time-in-the-country” RPs. The contents below reflect the opinion of experts in the field of radiopharmaceuticals and have been compiled for the benefit of the relevant stakeholders including both professionals and regulators. However, these views and opinions are not attributable to the individual expert's country or organisation. Some national authorities may have a different view or interpretation on certain aspects.

## Main text

### Distinct characteristics of radiopharmaceuticals—necessity of dedicated regulation

Radiopharmaceuticals require special regulatory consideration due to their distinct characteristics, which are outlined in this section.

#### Radioactivity and radiation safety requirements

Emission of ionizing radiation is the defining attribute of radiopharmaceuticals and their principal mechanism of action. Radionuclides with *gamma* or *positron* emissions are used for diagnostic purposes (photon emission), while therapeutic radiopharmaceuticals contain *beta* or *alpha* emitting radionuclides (charged particles).

The use of radioactive materials is strictly regulated in almost all MS. As a consequence, the production and use of radiopharmaceuticals are subject to a dual regulation from the authorities responsible for radiation protection and authorities responsible for pharmaceuticals or medicines. The requirements for the processes, work-flows and facilities from a radiation safety and a pharmaceutical safety point of view are contradictory in some respects. For example, the air pressure cascade for clean rooms where materials of high radioactivity are handled must address not just protection of the product, as is common in conventional pharmaceutical manufacturing, but also the radiation safety of the personnel in production and quality control areas. Another example is that sterility testing is constrained by radiation license requirements for transport to and sample processing in microbiology laboratories.

The radiation exposure of patients during diagnostic procedures is well below the threshold of deterministic harmful effects. Nevertheless, the radiation burden to achieve the required diagnostic image quality should be kept as low as reasonably achievable to minimise the risk for non-deterministic health effects. For therapeutic use, the cell killing effect of the radiation in tumours must be maximised, whereas the damage to healthy tissues and excretory organs must be minimised. Consequently, radiation dosimetry for organs and tissues is an important aspect in clinical trials as well as for routine clinical use (Konijnenberg et al. 2021). Pharmaceutical regulators are usually not familiar with dosimetry, radiobiology and imaging optimisation. In some countries, experts from radiation protection authorities support the pharmaceutical authorities in the assessment of these important aspects, or other external experts from academic or medical institutions are officially commissioned to assist.

Therefore, overlapping roles and responsibilities of the involved regulatory authorities require coordination, communication and streamlined requirements and processes to address these unique challenges smoothly.

#### Low amount of active pharmaceutical substance in patient dose

A great advantage of using radioactivity in medicine is the very low amount (often in the range of nanomoles) of active pharmaceutical substance required to supply sufficient radioactivity for the purpose. From a pharmaceutical risk-based point of view, the administration of a radiopharmaceutical is often far below the dosage required to produce any pharmacological effect (tracer principle). Therefore, chemical toxicity and pharmacological effects of radiopharmaceuticals are much less critical compared to conventional pharmaceutical substances (normally administered in the range of millimoles or higher) and are usually not pronounced at all. In addition, radiopharmaceuticals are typically applied only once or a few times in a patient’s life span for the intended investigation or treatment and in a highly controlled manner. This is reflected in substantially reduced requirements for pre-clinical and clinical testing in several guidelines (Korde et al. 2022).

In contrast, a small amount of active substance makes the synthesis processes vulnerable to the presence of even trace impurities in starting substances and materials (e.g. metal ions in metallic radionuclides) or to process deficiencies (e.g. incomplete drying during azeotropic evaporation, or slight variation in pH). Consequently, radiochemical reactions are extremely sensitive and must be performed under strict control and using starting materials of the highest quality. The materials are often prefilled in small single-use containers.

#### Short shelf life due to radioactive decay and radiolysis

The shelf life of radiopharmaceuticals is naturally limited by the physical half-life of the radionuclide. The medical radionuclides in use today have half-lives in the range of only a few minutes to approximately 10 days (European Pharmacopoeia), see Table 1. Most PET tracers decay completely in a few hours.

**Table 1:** Physical half-lives of some medical radionuclides for diagnostic and therapeutic use

Diagnostic use		Therapeutic use	
*Gamma* emitter		*Beta* emitter	
In-111	2.8 d	I-131	8.0 d
I-123	13.3 h	Lu-177	6.6 d
Tc-99 m	6.0 h	Y-90	2.7 d
*Positron* emitter		*Alpha* emitter	
F-18	110 min	Ra-223	11.4 d
Ga-68	68 min	Ac-225	10.0 d
N-13	10 min	Pb-212	10.6 h

The use of radionuclides of such short or very short physical half-life has medical reasons. For diagnostic purposes, a short half-life provides the intense radiation required during image acquisition to achieve good quality images in a reasonable time. All radioactivity remaining and decaying in the body after the completion of the diagnostic procedure would have no use and would result in unnecessary radiation burden; hence, the faster it decays, the better.

For therapeutic use, mainly in oncology by systemic administration, the physical half-life of the radionuclide must fit into the ranges of the biological half-life of the radiopharmaceutical in the tumours, organs and tissues, which is often shorter than a few days and rarely more than a week (Kassis 2008).

The shelf-life limitation due to the decay of the radioactivity can be partly counteracted by increasing the starting radioactivity; however, radiolytic degradation may limit this strategy, especially in the case of a sensitive biological vector. For physical, technical and radiation-safety reasons, the produced quantity of radioactivity also has its limits. Technical capacities have increased during the last few decades, but the starting quantity of radioactivity will remain a limiting factor for most radionuclides and radiopharmaceuticals.

Due to the medical advantages of short-lived radionuclides, they will continue to be preferred in clinical practice and, therefore, all challenges and limitations related to their handling and production will continue to exist. However, the extemporaneous on-site preparation of an RP that is necessary owing to the short-shelf life of most diagnostic radiopharmaceuticals (Hendrikse et al. 2022; Decristoforo and Patt 2017) also results in a reduction of the potential risk of microbial contamination, particularly when compared to conventional sterile injectable products that are usually stored for weeks, months or years.

#### Batches of small quantities and low volumes (small scale production)

Besides those mentioned above, there are several other factors that prescribe maximum batch sizes and volumes.

- The shipment of radiopharmaceuticals from external sources (commercial suppliers) is limited for various reasons, one being the very short half-life or shelf-life of the products. Due to this limitation, a major source of radiopharmaceuticals in routine practice is those prepared in-house in small quantities.
- Radiopharmaceuticals are often used for life threatening rare diseases not allowing accumulation of patients over longer periods of time. In cases of extemporaneous preparation, they are then often produced for a single patient only.
- The injection of a radiopharmaceutical (with the exception of some therapeutic applications) is usually performed as a short bolus for which the preferred injection volume is below 10 ml.
- The shielding requirements for handling the radiopharmaceutical in containers, syringes and automated injectors increase tremendously with increasing amount of radioactivity and volume.

As a consequence, only a limited number of patients are typically served with a single batch, and, at the same time, a very limited product volume may be available for quality control.

#### Absence of a classical stable “drug substance” that can be isolated

The classical pharmaceutical approach of separate manufacturing of

**Fig. 1:**
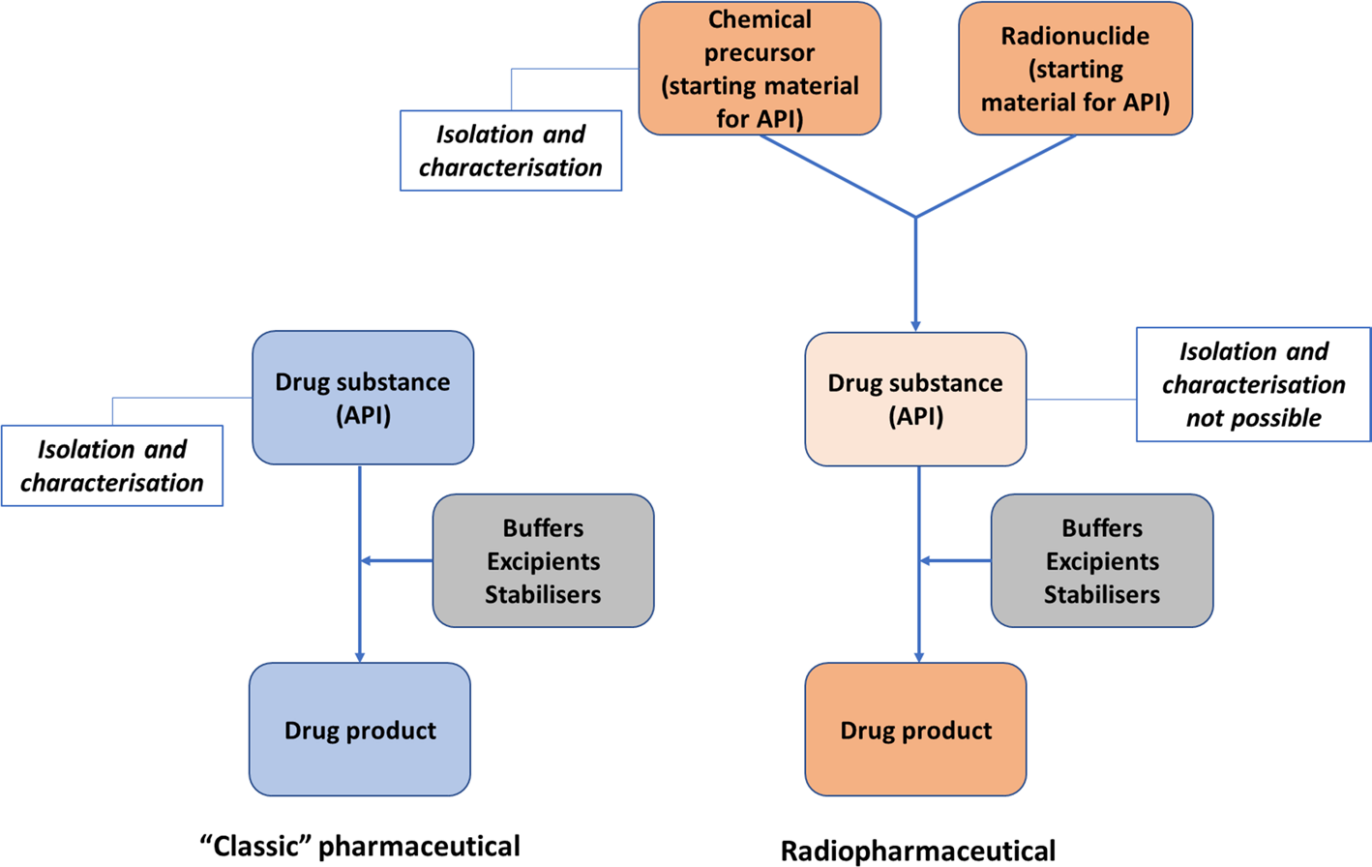
Comparison of the characterisation of drug substances and drug products in conventional (“classic”) medicines (left) versus in radiopharmaceuticals, where the drug substance cannot be isolated (right)

- A “drug substance” (also called active pharmaceutical ingredient, API) (Food and Drug Administration 2010) and
- A “drug product” (the final formulation containing the drug substance and additional substances such as excipients, stabilisers, solubility enhancers or buffers)

is hardly possible for radiopharmaceuticals, because the real drug substance, the radioactive molecule or ion, cannot be isolated, characterised and tested prior to formulation of the drug product (Fig. 1). Due to the very short shelf-life, the need to ensure effective radiation protection and other process-related reasons, the production is usually performed in a continuous and highly integrated manner.

Considering that the drug substance cannot be isolated and tested during radiopharmaceutical preparation, risk-based controls are usually applied at the level of starting materials or precursors, in-process controls and characterisation of final formulation to the extent possible.

Examples of drug substances, drug products and starting materials are given in Table 2.

**Table 2:** Examples of drug substances, drug products and starting materials

Drug Substance / Abbrev	Drug Product	Starting materials / precursors	
		Radioactive	Non-radioactive
[^99m^Tc]Tc-3,3-diphosphono-1,2-propanodicarboxylic acid ([^99m^Tc]Tc-DPD)	Aqueous solution of [^99m^Tc]Tc-DPD, SnCl_2_, buffer, + other excipients	Sodium ^99m^Tc- pertechnetate (Na[^99m^Tc]TcO_4_)	3,3-Diphosphono-1,2-propanodicarboxylic acid (DPD)
[^18^F]F-amino-3-[4-(2-fluoroethoxy)phenyl]propanoic acid ([^18^F]fluoroethyltyrosine; [^18^F]FET)	Aqueous solution of [^18^F]FET, EtOH, ascorbic acid, buffer, + other excipients	Aqueous solution of [^18^F]fluoride	O-tosyloxyethyl-N-trityl-tyrosine tert-butyl ester
[^68^Ga]Ga-Edotreotide ([^68^Ga]Ga-DOTA-TOC)	Aqueous solution of ^68^Ga- peptide, unlabelled excess of the peptide, ascorbic acid, buffer, + other excipients	^68^Ga-gallium chloride ([^68^Ga]GaCl_3_)	DOTA-TOC
[^177^Lu]Lu-Edotreotide ([^177^Lu]Lu-DOTA-TOC)	Aqueous solution of ^177^Lu-peptide, unlabelled excess of the peptide, ascorbic acid, buffer, + other excipients	^177^Lu-lutetium chloride ([^177^Lu]LuCl_3_)	DOTA-TOC

#### Pre-manufactured starting materials

For all the reasons outlined above, in-house preparation has a very important share in the supply of radiopharmaceuticals and may become even more significant (Hendrikse et al. 2022), see also Section "In-house production of radiopharmaceuticals, extemporaneous preparation". Many of such small-scale preparation activities take place with a high frequency, often daily or weekly in hundreds of sites worldwide. This demand has led to the evolution of commercial production of pre-manufactured ready-to-use starting materials for radiochemical synthesis and accompanying specialised small-scale automated equipment to simplify the on-site preparation of finished products. In the last 20 years, the industry has developed and introduced a number of innovative, dedicated solutions for radiopharmaceutical production, as outlined below:

- *Precursors of GMP quality*. They allow the reliance on the certified quality of the precursor for many of the essential quality control parameters for identity and purity.
- *Kits of GMP quality*. A precursor is pre-formulated in a vial with rubber septum together with excipients to allow a reliable labelling reaction by just adding a radionuclide-containing solution.
- *Cassettes and reagent sets of defined quality*. For more complex labelling processes, single-use cassettes and pre-filled reagents of defined quality have been developed. If they are combined with automated synthesis and dispensing systems, a high degree of standardisation and simplification is possible, ensuring patient safety with reduced requirements for quality control (for both incoming goods and end products).

Nevertheless, chemical precursors (Pijarowska-Kruszyna et al. 2021) and radionuclides (Neels et al. 2019) are not drug substances according to the common pharmaceutical definitions, but can be viewed as starting materials. Regulation should reflect this difference. Production according to GMP or a marketing authorisation (MA) for these material categories should not necessarily be a general requirement.

#### Quality control limitations and requirements

Considering that the drug substance cannot be isolated and tested during radiopharmaceutical preparation, characterisation and risk-based controls (e.g. bioburden) are usually performed at the level of chemical precursors and other starting materials.

Some standard compendial methods are difficult, or even impossible, to carry out on finished radiopharmaceutical products because the radionuclide half-life often limits the time available to complete all quality control tests before product release. It should be noted that, even with longer half-life radionuclides, radiopharmaceuticals may have similar limitations because of their shelf-life being shorter than their physical half-life. Radiation safety is also often a reason for substitution or post-release testing.

Where necessary and possible, alternative rapid methods are used (visual inspection and assessment of colour and clarity instead of instrumental determination of particulate matter, use of pH paper instead of pH potentiometry, rapid endotoxin testing, etc.).

In almost all cases, sterility testing for injectable radiopharmaceuticals cannot be completed prior to the administration of the product due to its high radioactivity, short shelf-life and small batch volume. This is reflected in general exceptions, e.g. in the European Pharmacopoeia (Council of Europe 2020) and in the regulatory provisions of other jurisdictions. Aseptic procedures, microbiological process validation and in-process controls (such as sterilising -filter integrity test) are used to ensure the sterile nature of the product to allow for preliminary release and retrospective sterility testing. In addition, use of pre-formulated kits (for simple reconstitution or compounding) and cassettes with prefilled reagents (for more complex syntheses) enables a closed system process and constitutes an important risk mitigation strategy against microbial contamination.

Testing for long-lived radionuclidic impurities requires allowing sufficient time to lapse for the main radionuclide to decay. Otherwise, it will be impossible to identify and quantify trace impurities due to the background radioactivity counts being too high, which results in the underestimation of impurities.

In principle, quality control (QC) tests, except for the sterility test, should be completed before the RP release. A post-release test must be justified with technical reasons, regulatory exceptions and a risk-based process validation. If justified, quality control parameters may be tested only during process validation and revalidation, e.g. radionuclide purity if a new batch of target material for irradiation is used.

### Quality and regulatory considerations based on the complexity of radiopharmaceutical preparation

As discussed above, radiopharmaceuticals are a unique class of medicinal products and in some aspects different to conventional drugs. In addition, from a regulatory standpoint, radiopharmaceuticals themselves can be divided into sub-categories based on:

- Type of application (diagnostic versus therapeutic)
- Type of drug development stage (first-in-human safety trials versus more advanced stages of clinical trials versus standard-of-practice clinical use)
- Type of preparation (kit-based radiolabelling versus complex radiosynthesis and purification)
- Intent to commercialise (to be placed on the market versus local use; industrial processes and volumes versus small-scale in-house preparation techniques)

The type of preparation in particular has considerable impact on regulatory requirements, in terms of:

- Facility requirements (e.g. manufacturing permission/authorisation)
- Type and extent of quality control methods
- Complexity and depth of quality assurance (QA) system
- Starting materials and precursors (quality, characterisation, testing)
- Training of staff
- Qualification of the personnel in charge of product release, management and general oversight

**Fig. 2:**
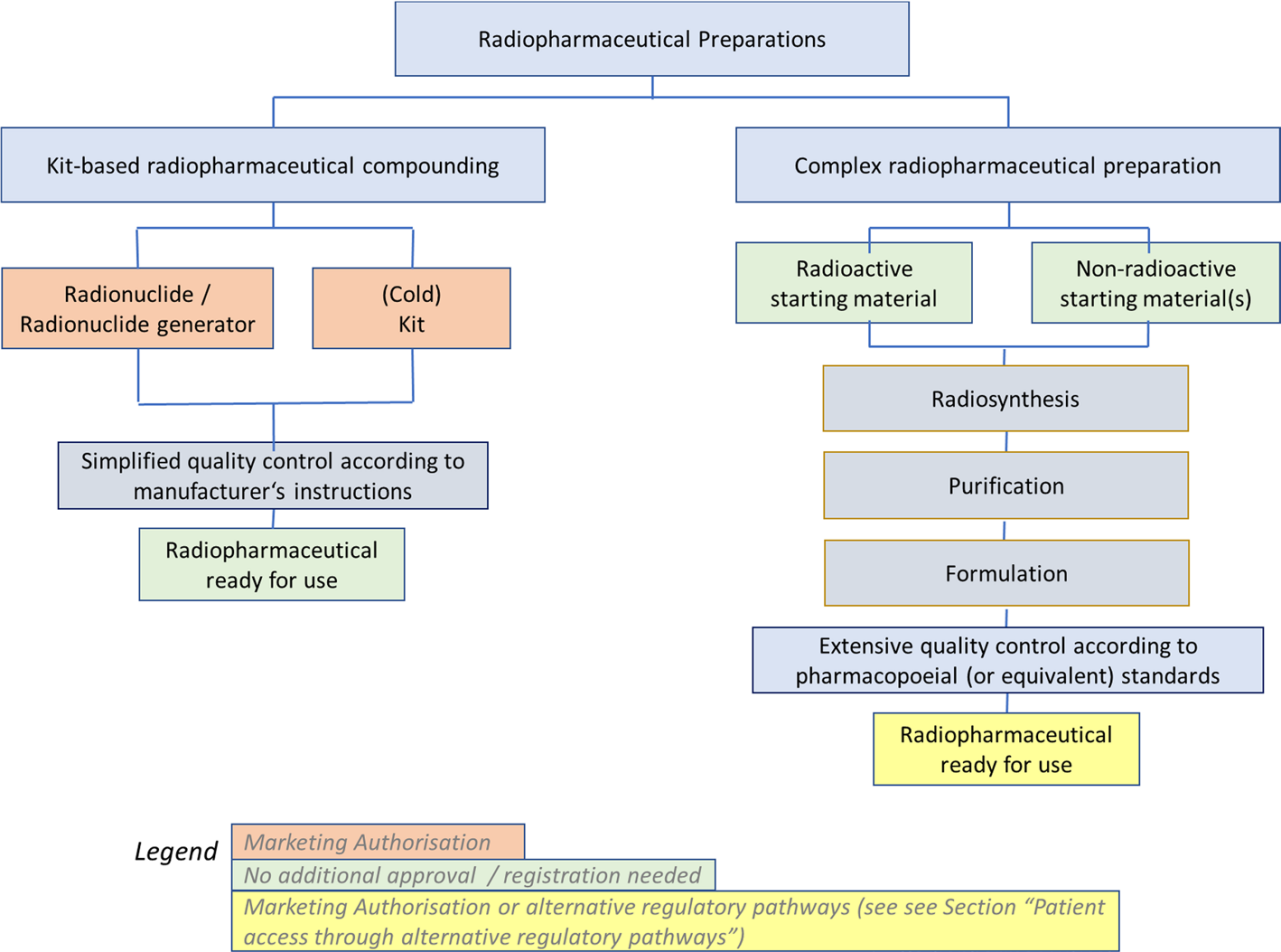
Two types of preparation of radiopharmaceuticals: kit-based radiopharmaceutical compounding (left) and complex radiopharmaceutical preparation (right). While kit-based radiopharmaceutical compounding is based on regulatory approval of radionuclides or generators and kits, for complex preparations the final product is subject to regulatory approval or other appropriate controls

Therefore, especially regarding in-house preparation, a clear differentiation between two distinct types of radiopharmaceutical preparation shall be made (see also Fig. 2):

- Kit-based radiopharmaceutical compounding
- Complex radiopharmaceutical preparation

A rationale and regulatory approaches, as well as other considerations resulting from this distinction, are given below.

#### Starting materials

In kit-based compounding, the starting materials are two approved medicinal products, usually one being a lyophilised mixture of non-radioactive substances (i.e. the kit for radiolabelling), including a biologically or physiologically active molecule binding the radionuclide, and the other a radionuclide generator eluate or another approved radionuclide solution as described in the kit package insert.

For complex radiopharmaceutical preparations, the starting materials can be many. As a minimum, a non-radioactive precursor for radiosynthesis, a radionuclide and a suitable solvent are required. Regulatory considerations with respect to radionuclides have recently been summarised (Decristoforo et al. 2022). Depending on the radionuclide production method and on the complexity of the synthesis and purification, a number of other chemicals, solvents and reagents may be needed. All these incoming chemicals are usually of a defined quality (identity and purity); GMP or pharmacopoeial grade chemicals are advantageous due to their well-defined quality and traceability but are not mandatory.

#### Radiolabelling or radiochemical synthesis

- Kit-based radiopharmaceutical compounding

A kit for radiolabelling is a non-radioactive pre-formulated combination of a precursor and suitable reagents that, once reconstituted or combined with a radionuclide solution, yields the final radiopharmaceutical drug product. Conventionally, kits are manufactured in a GMP-compliant manner and are supplied in a sterile form. In a kit for radiolabelling, a precursor is filled in a vial together with reagents and excipients to allow a reliable radiolabelling reaction by simply adding a radionuclide-containing solution, and simple additional steps such as brief heating or pH adjustment may potentially be required. This procedure is designed as an aseptic process (no further sterilisation required) and without purification steps. As a safety measure, a simple verification of the radiolabelling efficiency is required as a quality control step, which is described in the MA documentation and in the package insert or Summary of Product Characteristics (SmPC), and users are requested to follow these instructions. Of note, the kit concept is limited to simple radiolabelling reactions such as metal ion complexation reactions.

Kit-based radiopharmaceutical preparations can be carried out by hospital (radio)pharmacists or nuclear medicine technologists (or other professionals, as permitted by their scope of practice) in accordance with the instructions given in the accompanying product information such as the SmPC or package insert. This is the type of preparation that has been used historically from the beginning of Nuclear Medicine diagnostic applications (i.e. introduction of technetium-99m labelled radiopharmaceuticals).

In kit-based compounding, authorised or licensed kits are combined with authorised or licensed radionuclides (often eluted from a generator, either at the hospital radiopharmacy or at a central commercial radiopharmacy) in order to achieve the final radiopharmaceutical. In a true reconstitution process, the final product already exists in the vial and only addition of solvent or diluent is required. For kit-based compounding, the final medicinal product, which is the formulated-for-human-use radiolabelled compound, is generated in a radiolabelling reaction during the compounding process. However, from a microbiological point of view, the processes are alike. Kit-based preparations of radiopharmaceuticals are treated in a special way by current legislation in many countries, as such a procedure is considered to be a reconstitution or compounding rather than pharmaceutical manufacturing. Therefore, the final radiopharmaceutical, as well as the site of preparation, usually does not need additional approval or licensing when compounded radiopharmaceuticals are prepared for in-house use.

- Complex radiopharmaceutical preparation

Many radiopharmaceuticals, and in particular those containing fluorine-18 or carbon-11 for imaging with PET, are small organic molecules. Their preparation is much more complex, similar to multi-step organic synthesis. The production steps may include the radionuclide production in a cyclotron or elution from a generator, subsequent radionuclide purification, radiochemical synthesis, purification of the radiolabelled compound, formulation, sterilisation by filtration and dispensing. The radiosynthesis reaction (binding of the radionuclide to the precursor) is designed to be as quick and simple as possible. Precursors are typically synthetic molecules and are often produced in a manner similar to an active pharmaceutical ingredient. To reduce the risk of producing radiolabelled by-products and not being able to identify these during quality control testing, the number of reagents used in a radiosynthesis is limited, the chemical transformation carefully designed and controlled and procedures are performed by skilled and experienced personnel. During purification steps, unreacted chemicals and solvents incompatible with administration to humans are removed, and the product is formulated in a suitable matrix, often in 0.9% saline with or without stabilisers.

Complex radiolabelling procedures, which may involve a multi-step synthesis and purification, are often carried out using pre-programmed and automated radiosynthesis and filling equipment that use purpose-designed fluid paths by employing single-use cassettes and pre-filled reagents of defined and controlled quality. While the initial aim of these remotely-controlled systems was to avoid manual handling of large quantities of radioactive materials for the sake of radiation protection, they also allow a significant degree of standardisation and traceability.

In contrast to kit-based radiopharmaceutical compounding, starting materials for complex radiopharmaceutical preparations are not as such subject to an approval or recognition process by a competent authority. However, the final labelled product (i.e. the radiopharmaceutical) should be subject to some kind of regulation (see Section “Patient access through alternative regulatory pathways”). The exact type of regulation differs among the individual countries. However, a common approach is that the product should at least be described in a local or international pharmacopoeia, thus ensuring that the product itself is scientifically established and has proven clinical efficacy and benefit for the patient.

#### Formulation

Kit-based radiopharmaceuticals are pre-formulated and therefore used as such; the radiolabelling reaction is usually quantitative. The preparation is rejected if quality control test results show insufficient radiolabelling efficiency (incomplete radiolabelling).

During complex radiopharmaceutical preparation, purification of the radiolabelled compound is usually mandatory and may consist of more than one purification step before formulation. The radiochemical yields in these types of radiolabelling reactions are seldom quantitative. Therefore, the compound of interest usually needs to be separated from unreacted starting materials, by-products and organic solvents incompatible with physiological conditions. After this, the product is typically sterilised (usually by filtration) and formulated.

#### Quality control

For kit-based products, the manufacturer of the kit prescribes quality control procedures in the accompanying product information (SmPC, package insert, etc.). The testing is usually limited to simple non-instrumental techniques, those that are feasible in a pharmacy or a nuclear medicine department.

For complex radiopharmaceutical preparations, extensive quality control according to pharmacopoeia or equivalent standards is required. The tests employ different analytical techniques, often using dedicated analytical instrumentation (high-performance liquid chromatography, gas chromatography, gamma spectrometry, etc.), to assess the parameters that are common for any conventional non-radioactive drugs (e.g. residual solvents, chemical purity), as well as those that are attributable to the radioactive nature of this special type of pharmaceuticals (e.g. radiochemical purity, radionuclidic identity and purity).

#### Documentation

For kit-based products, the documentation of the compounding itself can be restricted to a control of the starting materials (i.e. kit and radionuclide or generator) and a preparation log with minimum information required to trace back the individual patient dose or application to the corresponding preparation, such as date and time of the preparation, operator, lot numbers and results of quality controls.

For complex radiopharmaceutical preparations, a documentation system should be established as part of an overall quality assurance system (e.g. Gillings et al. 2021). Risk assessment of critical quality parameters, including starting materials, should be carefully considered and documented. Concrete mitigation strategies should be developed to avoid product contamination and ensure product integrity and quality.

Some features of the laboratories or buildings where the production takes place, as well as personnel qualification and behaviour, can also impact product quality, and should therefore be included in the quality assurance documentation.

#### Testing laboratories and pharmacopoeias

In some countries, national pharmaceutical quality control laboratories support national medicines regulatory authorities to verify that active pharmaceutical ingredients, excipients and pharmaceutical products meet the prescribed specifications (World Health Organization 2014).

In the case of radiopharmaceuticals, it is very difficult and of limited effectiveness to set up a centralised facility for analytical testing of the fast decaying finished products. Quality control methods for radiopharmaceuticals include tests which are different from those for conventional drugs, requiring very specific equipment and techniques (e.g. gamma spectrometry), highly specialised personnel and a licence from a national nuclear and radiation safety regulatory body. In addition, many pharmacopoeias, including The International Pharmacopoeia (Ph. Int.) published by the WHO (The International Pharmacopoeia 2023), contain general sections on radiopharmaceuticals that describe testing methods, as well as monographs for specific radiopharmaceuticals. These sections, together with general chapters on the standards for raw materials, intermediates and finished medicinal products, can be used as the scientific basis for quality control and quality assurance of radiopharmaceuticals during their development and production. When a pharmacopoeia monograph is available, radiopharmaceuticals should be produced to meet the specifications described within it. For many countries, national pharmacopoeias are legally binding. The Ph. Int. is more recommendatory in nature, but it serves as a benchmark for all countries. For the Ph. Int., the monographs on radiopharmaceuticals were developed in collaboration with the IAEA.

Local regulatory oversight, including site visits to observe workflows and processes at the production facility may provide sufficient assurance that ingredients and finished products will meet the required specifications. If the existing national procedures still require independent testing for registration or authorisation of a radiopharmaceutical, expertise and infrastructure of the laboratories already existing in many countries can be considered for such testing purposes after ensuring the absence of a conflict of interest. This will be similar to the technical support organisations used by many national nuclear or radiation safety regulatory bodies (Global Nuclear, IAEA 2023).

Another useful approach is that for products that have MA in the country of origin, testing as part of the authorisation process for importation should not be strictly necessary. If required, sharing of original documents for review can be requested. This becomes particularly relevant due to mutual agreements and cooperation between the authorities of different regulatory jurisdictions. Adoption of this model as a “good practice” (i.e. avoidable repetition of testing) should be encouraged for enhancing ease of availability of RPs in a large number of countries. In conclusion, a national testing laboratory for radiopharmaceuticals may be replaced by other supervision concepts, as already implemented by some countries.

### Personnel requirements

Personnel requirements for radiopharmaceutical production will depend on the workload and nature of the production method. In all cases it is important that appropriately trained and experienced persons take responsibility for quality assurance, production and quality control (PIC/S 2014). These may not necessarily be pharmacists, depending on the expertise required—e.g. production of more complex products may require in-depth knowledge of radiochemistry, while radiopharmaceutical production from kits may be undertaken by suitably qualified nuclear medicine technologists. Regulating authorities may have to consider exceptions to the requirements set for production of conventional medicines. Allowing qualified nuclear medicine technologists to prepare radiopharmaceuticals from kits using closed procedures is a safe and appropriate approach that allows access to and availability of radiopharmaceuticals at small centres or in countries with a limited number of radiopharmacists.

Training should specifically address radiopharmacy knowledge and include, amongst other factors, radiation safety, radiochemistry, aseptic techniques, radioanalytical methods and quality management (World Health Organization 2014, 2010). At least the person who oversees the whole production process and is responsible for the release of radiopharmaceuticals (e.g. Responsible Person or Responsible Pharmacist) should have a suitable specialist qualification and significant hands-on experience. Examples of qualifications offered include the Postgraduate Certificate Course in Radiopharmaceutical Chemistry/Radiopharmacy of the European Association of Nuclear Medicine (EANM) (EANM 2020), the recently launched the Society of Nuclear Medicine and Molecular Imaging’s Qualified Systems Personnel Training Program (SNMMI 2022) and the Australian Training, Education and Assessment Program (Australasian College and of Physical Scientists Engineers in Medicine 2023).

For manufacturing of products in relatively large scales for the open market (e.g. radionuclide generators and kits), several highly qualified persons are required to take separate responsibility for overall quality assurance, production and quality control. Where the facility is small due to low demand for products, it may not be economically feasible to employ separate deputies for each of the above positions. Some measure of taking over responsibility across different functions may be required, e.g. appointing an individual who can act as deputy for more than one of the functions at different times. The nature of the work, scheduling of tasks, and batch sizes should be taken into account. However, for each production batch, it remains important to separate the responsibility for production (i.e. synthesis) and quality control.

For in-house, small-scale production, the number of personnel that can be employed will necessarily be low, and for the preparation of RPs from licensed kits and generators often only one person performs the tasks. It is, however, still essential that the responsibilities for production and release are clearly defined, even in small units (PIC/S 2014).

It is likely that mandatory stipulations for personnel qualifications in pharmaceutical production may not yet exist in some countries. It is desirable to institute eligibility requirements that personnel should meet, including those with managerial responsibilities, along with creating schemes for the staff to obtain formal qualifications and/or certification. Training and refresher or update events may be necessary and the IAEA may consider providing suitable online and physical support in this context.

### Requirements for manufacturing practices and quality assurance

In the production or preparation of any pharmaceuticals, including RPs, manufacturing aspects are always of paramount importance, as they provide the necessary safety and traceability framework, which ultimately guarantee that radiopharmaceuticals are prepared with characteristics suitable for the intended purpose. In many countries, the above framework is well known under the name of Good Manufacturing Practice (GMP), which can be defined as “*A set of practices, using a traceable process, which ensures that radiopharmaceutical products are consistently produced and controlled to the quality standards appropriate for their intended use and designed to consistently yield the radiopharmaceutical product. Good Manufacturing Practices fall under the umbrella of the overall Quality Management System*” (European Commission 2017). This definition embraces all the aspects of RP preparation, from recommendation on practical aspects, such as premises and facilities, equipment, production and in-process and quality control aspects, to other quality assurance related topics like personnel, documentation, self-inspections, etc. Industrial manufacturing of RPs is highly regulated in many countries, including a GMP framework defined by a set of guidelines, often legally binding.

In case of non-industrial, “in-house” preparation of RPs, adherence to “industrial” GMP may not be required, and implementation and extent of the Quality Management system may be defined, based on a risk assessment of the practice, as is also recommended by a resolution of the Council of Europe for pharmacy preparations (Scheepers et al. 2017). For radiopharmaceuticals, such a risk assessment should be based on the following criteria:

- The degree of drug development, i.e. whether the RP is a well-established, routinely used RP (e.g. [^99m^Tc]Tc-MDP for bone scans, [^18^F]FDG) with clear clinical indications or an investigational RP, whose in vivo characterisation is still ongoing and production process may be at a preliminary stage of development;
- The complexity of the preparation, which may range from simple compounding of a kit with the intended radionuclide, which may eventually be prepared manually, to complex multistep radiosynthesis processes, whose preparation requires automated systems, may include purification apparatus (e.g. semi-preparative HPLC) and may make use of multiple reagents or solvents, which increase complexity of quality controls;
- The type of RP of concern, i.e. whether the RP is to be used for diagnostic vs therapeutic purposes; different types of RP preparations may require specific technicalities, which in turn impact quality assurance implementation; for instance, quality control equipment and procedures in the case of RPs labelled with *α*-emitters are quite different from those typically used in the quality control of γ or *β* emitting radionuclides;
- The production scale, i.e. whether the RP preparation is intended for use with a limited number of patients, possibly within the same healthcare establishment where the RP is prepared (in-house preparations), or a large scale production, aimed to provide doses for a large number of patients, located in the same or in different hospitals; the latter entails the distribution of the RP which, irrespective of commercial or non-commercial production, poses additional safety issues;
- Additionally, the microbiological risk should be considered and should prompt an increased frequency of testing in case of preparations considered to be at high risk (e.g. cell labelling);
- Even the half-life of the intended radionuclide may impact the general design of QA framework, e.g. due to the challenge in performing quality control before batch release in case of short or very short half-lives, that further strengthens the need for a robust QA system;
- Radiation protection aspects, although they are not intrinsically to be considered as related to quality assurance, are always of concern and may have many impacts, from the design of the HVAC system, where potentially opposite requirements may arise and need to be properly implemented and harmonised to avoid potential conflicts, to the design of production and dispensing hot cells, which also have to provide suitable shielding for the operators while at the same time ensuring microbiological protection to the product.

The framework of GMP is a very well-established concept in the field of pharmaceuticals, and many guidelines are publicly available. Although these guidelines were originally made to provide guidance to the conventional pharmaceutical industry, over the years they became more and more tailored for the preparation of radiopharmaceuticals and, at least in part, specifically address their uniqueness. For instance, dedicated chapters and/or full guidelines may be found in EU (European Commission 2017), US (U.S. Food & Drug Administraion 2023; FDA 2018) GMP guidelines, guidelines from WHO (World Health Organization 2014) and the Pharmaceutical Inspectors Co-operation Scheme (PIC/S) (PIC/S 2023a, b). In 2014, PIC/S released a document aimed to provide guidance for the preparation of medicinal products in healthcare establishments (PIC/S 2014), which includes an annex specifically dedicated to the preparation of RPs. Moreover, scientific associations such as the European Association of Nuclear Medicine issued several guidelines that cover both general preparation of RPs on a small scale (Gillings et al. 2021) and more specific aspects, such as the preparation of an Investigational Medicinal Product Dossier (IMPD), quality risk management or the validation of processes and analytical procedures (Gillings et al. 2021, 2020, 2022; Todde et al. 2014, 2017). Although not legally binding, they provide useful recommendations that might be considered in the implementation of an appropriate quality assurance setting. Finally, the IAEA, in collaboration with the WHO, have recently published, or are in the process of publishing, updated guidelines focused both on general GMP principles for RPs (World Health Organization 2020) and dedicated to the preparation of more specific types of RPs, such as investigational RPs (World Health Organization 2022) or the in-house production of “cold” kits (WHO, in progress). This comprehensive body of documentation, summarised in Table 3, including the present document, combined with a judicious risk analysis, provide a potential RP manufacturer with the necessary information for implementation of their own QA/GMP system to the satisfaction of concerned regulatory authorities.

**Table 3:** Relevant guidelines and texts in relation to GMP for radiopharmaceuticals

Guideline/Text	Origin/ Organisation	Topic	References
*General GMP guidelines*			
PE 009-17 Guide to Good Manufacturing Practice for Medicinal Products Part I and Annexes (including Annex 3 on “Manufacturing of radiopharmaceuticals”)	PIC/S	General GMP guidelines	PIC/S (2023a, b)
WHO Good Manufacturing Practices	WHO	General GMP guidelines	World Health Organization (2014, 2010)
Current Good Manufacturing Practice (cGMP) Regulations	FDA	Official US GMP guidelines	U.S. Food & Drug Administraion (2023)
Volume 4 of "The rules governing medicinal products in the European Union": Good Manufacturing Practice (GMP) guidelines (including Annex 3 on “Manufacturing of radiopharmaceuticals”)	EU	Official EU GMP guidelines	European Commission (2017)
*Specific GMP guidelines for radiopharmaceuticals*			
International Atomic Energy Agency and World Health Organization: guideline on good manufacturing practices for radiopharmaceutical products	IAEA/WHO	General GMP guidelines on RP preparation	World Health Organization (2020)
PET Drugs – Current Good Manufacturing Practice	FDA	GMP specific for PET RP manufacturers	FDA (2018)
PE010-4. Good practices for the preparation of medicinal products in healthcare establishments	PIC/S	GMP recommendations for pharmaceuticals (including RPs) prepared in hospitals	PIC/S (2014)
Guidelines on current good radiopharmacy practice (cGRPP) for the small-scale preparation of radiopharmaceuticals	EANM	cGRPP for the small scale preparation of RPs	Gillings et al. (2021)
IAEA/WHO guideline on good manufacturing practices for investigational radiopharmaceutical products	IAEA/WHO	GMP for investigational RPs	World Health Organization (2022)
IAEA/WHO guideline on good manufacturing practices for in-house cold kits for radiopharmaceutical preparations	IAEA/WHO	GMP for in-house prepared cold kits for RP preparations	In progress
*Other documents*			
EANM guidelines for the preparation of an investigational medicinal product dossier (IMPD)	EANM	Guideline for the preparation of an IMPD, including practical examples	Todde et al. (2014)
Guidelines on Radiopharmaceuticals	EMA	EU guidelines for MA applicant	EMA. European Medicines Agency (2018)
Resolution CM/Res(2016)1 on quality and safety assurance requirements for medicinal products prepared in pharmacies for the special needs of patients	Council of Europe	Requirements for the preparation of medicinal products in pharmacies	Scheepers et al. (2017)

### Regulatory authorities: interfaces, communication and training

#### Interface between pharmaceutical and radiation safety regulations

The evaluation of safety and efficacy of radiopharmaceuticals, and approvals for clinical use, requires a regulatory approach that incorporates a coordinated evaluation of both the production and pharmacology of the radiopharmaceutical and the radiation safety aspects of production and patient use. Pharmaceutical regulators and radiation safety regulators should cooperate, possibly by forming joint committees, for the evaluation of production premises or facilities and of applications for MA or other forms of permission for production. Only a combined approach can ensure that apparently conflicting guidelines for radiation safety and pharmaceutical production are appropriately interpreted and implemented.

The use of radionuclides requires an appropriate and practical regulatory approach to the evaluation of the pharmacological properties of radiopharmaceuticals, considering their short half-lives and trace amounts. This approach should apply to both routine production and use of radiopharmaceuticals, and for the evaluation of radiopharmaceuticals in clinical trials.

#### Other authorities

It is important that all types or pathways of radiopharmaceutical production are regulated, to ensure that all products are safe and effective. In some countries, certain aspects of radiopharmaceutical preparation, like kit compounding, may not be subject to regulation by the health or pharmaceutical regulatory authority but are regarded as falling within the scope of practice of pharmacy and are therefore under the control of a professional association such as a pharmacy council. If this is the case, regulation of such facilities should not be neglected, but be overseen by the appropriate authority. Additionally, the professional council may add some valuable views regarding professional responsibilities in the whole chain of development and production of radiopharmaceuticals, together with the National Health and Radiological Regulatory Bodies.

#### Radiopharmaceutical regulator qualification and communication pathways

To ensure efficient regulation of radiopharmaceutical production, the persons responsible for regulation must possess both regulatory training as well as expertise in radiopharmaceutical development and/or manufacture. Intimate knowledge of the radiation emitting drugs’ unique characteristics is essential to make well informed decisions. The regulators may obtain this expertise through a professional background (i.e. regulators that have formerly been trained and have been involved in radiopharmaceutical development and production) or through having successfully completed a defined training program on the principles governing the unique characteristics of RPs. Regulators should also have a legal foundation to consult with independent expert groups in situations where the specific matters are outside of the regulator’s primary expertise. Creation of a standing expert panel for such assistance and guidance may be considered to strengthen or augment the regulatory practices at national level. It should be emphasised that while the regulator qualification requirements described above apply to medicinal agencies with subspecialty in radiopharmaceuticals, in some regions of the world the responsibilities for radiopharmaceutical regulation may be relegated to agencies that are normally responsible for general radioactive material handling, licensing and safety, irrespective of use. In those instances, the regulators should consider adopting the recommendations provided in this guidance, in addition to their responsibilities related to safe handling of radioactive materials, and cooperation with the medicines regulatory authority is recommended.

Established mutual communication mechanisms between the regulators and producers are also absolutely essential for effective regulation of radiopharmaceuticals for human use. These communication pathways should be in the form of official communications and meetings between the regulators and a particular producer for the purpose of addressing a specific question, as well as in the form of periodic meetings between regulators and the producer community for the purposes of providing feedback and information exchange. The ability to have two-way communication between the regulator and the producer provides several key advantages to both parties. Individual meetings with the regulators allow the producer to receive clear regulatory input on questions that may not be adequately addressed in the existing regulations and guidance. Most importantly, all these communication pathways yield regulators possessing the necessary professional knowledge and understanding that enables them to make well-informed decisions. Such decisions, based on appropriate risk assessment, facilitate patient access to radiopharmaceuticals, while maintaining the appropriate measures of quality.

### Facilitation of patient access to radiopharmaceuticals through the marketing authorisation regulatory pathway

Regulatory mechanisms for access to radiopharmaceuticals, including MA, should ensure timely patient access to safe and effective medicines and should be fit for purpose. The uniqueness of radiopharmaceuticals requires various licensing pathways to protect public health and ensure patients receive safe and effective diagnosis or treatment.

#### Marketing authorisation overview

As with all drugs, the MA for radiopharmaceuticals represents an approval, granted by the designated medicines governing agency in a specific region, to sell the drug so that it may be used in standard clinical care for a specific regulatory-approved indication. The essence of MA is that the radiopharmaceutical can be sold for profit (i.e. marketed). The most challenging aspect of obtaining MA is for the producer, unless its clinical use is already well established, to clearly demonstrate to the regulators the radiopharmaceutical safety and efficacy for the proposed indication, by conducting a series of clinical trials in a specific patient population with a specific disease or medical condition.

From the regulatory standpoint, approved product labelling and drug quality are the two additional components that are essential for obtaining MA. Product labelling includes all documentation which provides instruction to clinicians on how the drug should be used in patients, ensuring standardisation.

Product labelling not only includes the immediate container label, but also all of the accompanying instructional documentation such as package inserts in the US or SmPC in the EU. This standardisation helps to ensure that radiopharmaceuticals are used appropriately by healthcare professionals and patients.

The drug quality aspects of MA for radiopharmaceuticals are similar to those for other medicines but with some additional considerations due to their radioactive nature. As stated in Section "Regulatory Authorities: Interfaces, communication and training" of the present position paper, radiopharmaceuticals require a fine-tuned balance between procedures or controls aimed at ensuring quality, and procedures or controls ensuring radiation safety and handling.

Marketing authorisation can be granted to completely new agents with novel approved indications, generic radiopharmaceuticals which are essentially copies of already approved drugs and pharmaceutically equivalent radiopharmaceuticals, which have minor differences in formulation from the already approved drug that do not alter safety and efficacy. The MA approval process is comparatively long, costly and resource demanding. However, in some circumstances, certain regulatory provisions should be established that can reduce the burden on MA applicants in order to facilitate patient access to MA approved radiopharmaceuticals, which are described below.

#### Local or regional marketing authorisation for generic and pharmaceutically bioequivalent products

Analogous to all drugs, generic and pharmaceutically bioequivalent radiopharmaceuticals offer several key benefits in regard to improving patients’ access to medicines. In the context of this guidance, the biggest advantage of implementing the MA regulatory pathway for the domestic manufacture of generic and pharmaceutically bioequivalent radiopharmaceuticals is that it allows physical patient access to drugs in certain regions of the world where the drugs manufactured by the respective primary MA holders cannot be practically delivered. Unlike traditional pharmaceuticals, radiopharmaceuticals do not enter the drug distribution chain because of their relatively short shelf-lives of several hours to several days. Due to these constraints, they must be supplied directly from the production facility to the authorised end-user, and the international supply is extremely challenging. Granting local MA for regional production and supply addresses these challenges. Another significant advantage of establishing generic or pharmaceutically bioequivalent local radiopharmaceutical supply is the significantly reduced cost of having access to these agents. The premise for having such a supply is the availability of a proven measure of safety and efficacy for a specific clinical indication, which ideally exists in the form of previously approved MA for the same drug product in other countries. Therefore, repetitive clinical trials demonstrating clinical safety and efficacy may not be required, thus saving resources and time and greatly facilitating integration of the agent into standard clinical care at a reduced cost. Therefore, once implemented, the domestic or regional manufacture of such radiopharmaceuticals is beneficial to patients by improving access. Lastly, another potential benefit of establishing domestic production is increasing regional economic growth, driven by domestic drug manufacturing entities.

Given the unique nature of radiopharmaceuticals, the regulatory mechanisms applied for the approval of generic radiopharmaceuticals and pharmaceutical equivalents may require several special considerations that, based on appropriate risk assessment, provide adequate balance between product quality and allowing patient access to these drugs.

In a first place, the process for granting local MA approvals for domestically produced radiopharmaceuticals that rely on the prior findings on safety and efficacy of the referenced drug products should be clearly defined and should be made in consultation with the national and/or regional multidisciplinary professional groups, including medical doctors who will be responsible for using the drug in patients.

Next, as the field of molecular imaging and radionuclide therapy develops, relevant regulatory recommendations need to be established.

Lastly, while region-specific MA radiopharmaceutical regulations may be promulgated by either a division of the regulatory body responsible for regulation of all medicines in the region or a by a standalone regulatory entity specialising only in the regulation of radiopharmaceutical production, the considerations that must be taken into account when generating and implementing the regulations governing access to MA radiopharmaceutical remain the same.

From the regulatory point of view, the more critical decision to be taken by the regional regulators is whether the local MA application requires additional clinical trials. In order to make that determination, the regulators first need to determine whether the radiopharmaceutical named in the MA application qualifies as either a true generic (i.e. the final drug product formulation is a duplicate copy of the referenced drug product formulation) or a radiopharmaceutical that could be considered pharmaceutically bioequivalent (i.e. some differences in inactive ingredients between the proposed drug product formulation and the referenced drug product formulation do exist, but these differences are minor and, based on provided scientific evidence, will not affect the drug’s safety and efficacy when used clinically in an identical manner as the referenced drug).

In the case of reviewing an MA application for generic radiopharmaceuticals, the regulators should focus on ensuring that the proposed drug product formulation is qualitatively and quantitatively identical to the referenced drug. In other words, both drug product formulations should contain the same active ingredient and all of the inactive ingredients in the same amounts. In addition, the regulators should ensure that the proposed drug product will have the same clinical indication and will be used in the clinic in an identical manner to the referenced drug. As long as these requirements are satisfied, no additional clinical trials should be required for generic radiopharmaceutical MA application approval.

In case the proposed drug product differs from the reference drug product with respect to inactive ingredients such as buffers, pH adjusters and antioxidants, it may still be deemed to be pharmaceutically bioequivalent, not requiring additional clinical studies for approval. However, pharmaceutical bioequivalence should be demonstrated by additional suitable non-clinical studies, also known as “pharmaceutical cross bridging” studies, that may take advantage of existing scientific literature to support the absence of impact on physicochemical properties from the differences in formulation. The primary studies need to demonstrate pharmaceutical bioequivalence should be in the form of conducting chemical bridging experiments and demonstrating that the difference in inactive ingredients between the proposed drug product and the referenced product does not alter the physicochemical properties, and therefore the pharmaceutical equivalence, of the drug. Additional studies, which may or may not be required, include additional in-vitro studies demonstrating pharmacodynamic equivalence, and/or animal in-vivo studies demonstrating pharmacologic equivalence. The extent of the required number and the types of studies should be decided on a case-by-case basis. In particular, flexibility should be allowed in terms of the types of antioxidants (also known as radioprotectants, radical scavengers or radio-stabilisers) that may be used in the formulation for autoradiolysis protection and stabilisations. For example, substitution of radioprotectants with well-known radiopharmaceutical antioxidant properties, such as ethanol, gentisic acid, ascorbic acid or a combination thereof, should only require chemical bridging studies to demonstrate that the physicochemical properties are unchanged and that the two products are pharmaceutically equivalent. Once the pharmaceutical bioequivalence has been established through “cross bridging”, the same principles of relying on prior findings of safety and efficacy as for generic radiopharmaceuticals apply.

#### Marketing authorisation with orphan drug designation

Orphan drug designation is granted to MA applications for new, generic or pharmaceutically bioequivalent radiopharmaceuticals intended to treat rare diseases. Due to the limited patient population, it may be challenging to meet the requirements for full MA, or companies may have little financial incentive to invest in developing the products or marketing such products in specific markets. In some jurisdictions, the orphan drug designation provides the MA applicant incentives such as tax credits, reduced licence fees, research grants, and extended exclusivity periods to encourage the development of drugs for rare diseases; the extent of documentation requirements, however, usually remain unchanged. The orphan drug designation benefits patients with rare diseases as well, increasing the likelihood that treatments will be developed for their condition. The regulatory authorities normally decide whether the orphan drug designation may be granted and may indicate the conditions for which orphan drug designation is applicable. For example, regulators may set a limit on specific disease prevalence which would need to be met in order to qualify for this designation.

#### Import authorisations for radiopharmaceuticals with marketing authorisation in other regions or countries, but are not approved locally

In some cases, the MA holder or manufacturer may not register their products in all countries due to commercial reasons or small market sizes for specific products, especially in less developed regions and countries. Therefore, to ensure patients can still access known or proven radiopharmaceuticals registered and available in other countries, there should be a mechanism to facilitate special use permits or authorisation from the regulatory authority that allows the importation and use of radiopharmaceuticals for a specific purpose or patient population. This option may require the demonstration of a clear medical need and provision of information about the safety and efficacy of the product or registration in other countries. In these cases, the healthcare professional should ensure proper record-keeping, monitoring and reporting of adverse events to the regulatory authorities. This mechanism should not be applicable in situations where a product is locally registered and an available or viable alternative exists.

#### Marketing authorisation—conclusion

Once established, the MA pathway provides a robust mechanism for patient access to safe and effective radiopharmaceuticals. However, it is important to consider that in many regions of the world, MA is not the sole regulatory mechanism that allows the use of the radiopharmaceuticals as part of standard clinical care. Under certain circumstances, specific radiopharmaceuticals may be used to diagnose and treat patients following an alternative licensing pathway that has been established and approved by the local or regional medicines regulating agencies. The purpose of these alternative pathways is to ensure the best possible access of patients to these medicines while maintaining appropriate quality. Some examples of these alternative pathways are provided in the next section.

### Patient access through alternative regulatory pathways

#### In-house production of radiopharmaceuticals, extemporaneous preparation

Even though for many radiopharmaceuticals MA is the preferred regulatory pathway, its implementation may be both cost and resource prohibitive under certain circumstances. Challenges may include limited available regulatory infrastructure and resources for such implementation in certain regions or countries, as well as limited patient access to MA approved agents due to very short shelf-life and geographical limitations. Another challenge is a lack of economic viability for establishing a regional supply of MA approved radiopharmaceuticals simply due to the small number of patients requiring a certain radiopharmaceutical. Novel approved products in particular are often only made available in major markets and therefore not available for many years in developing markets. Under such circumstances, the radiopharmaceutical should be produced for local use utilizing a practice known as “in-house” production. Filing of a MA for in-house produced RPs should, therefore, not be required. However, a mechanism must exist where the local health regulatory authorities must be notified of, or approve of, the producer’s intent to produce radiopharmaceuticals in-house for patient use.

This application to use “in-house” produced radiopharmaceuticals in patients could be in the form of a simple document stating that the respective radiopharmaceutical already has a MA elsewhere, which satisfies the safety and efficacy requirement, and is of equivalent quality to the referenced drug. Alternatively, the application may state that use of the radiopharmaceutical is justified based on the fact that it follows the established pharmacopoeial requirements recognised by local regulators. Moreover, in certain cases the use of radiopharmaceuticals may be justified even in the absence of prior MA or pharmacopoeial monographs—e.g. when the use of a radiopharmaceutical in a specific patient population is well-established by the local medical community or when a medical doctor decides to treat a specific patient with a radiopharmaceutical based on professional judgement. The regulator’s decision to grant approval should be in consultation with the relevant local medical doctor professional groups, and the use of the in-house produced radiopharmaceutical in a given patient should be as per request of the ordering medical doctor (similar to the magistral formulae approach). Communication mechanisms between the regulators and producers should be established, and the producers may be subject to periodic audits by the local regulators.

The “in-house” produced radiopharmaceuticals may be produced either within a hospital or a clinic for internal use or within a local production facility to be distributed to local hospitals or clinics. The overall responsibilities for the production and use in humans have to be clearly defined, including the responsibilities of the ordering medical doctor. Currently, the requirements for in-house production of radiopharmaceuticals vary depending on the regulatory requirements in the country where the production occurs. In many countries, this process is based on the pharmacy regulations in the respective jurisdiction and is usually performed under the umbrella of the hospital pharmacy. In others, hospitals and clinics must have a dedicated license or permit to produce radiopharmaceuticals and adhere to strict quality and safety standards. Some examples of specific regulations can be found in a publication of Decristoforo et al. (2017).

#### Additional advantages of in-house preparation of radiopharmaceuticals

- Personalised patient treatment

One of the main purposes of in-house production of radiopharmaceuticals is to provide patients with access to customised and timely radiopharmaceuticals for diagnostic and therapeutic purposes. In-house production of radiopharmaceuticals means that the radiopharmaceuticals are produced on-site at a hospital or clinic rather than being outsourced to a third-party manufacturer. Therefore, in-house production of radiopharmaceuticals can benefit patients, allowing for greater flexibility and customisation in their treatment. For example, radiopharmaceuticals can be tailored to the specific needs of individual patients, such as adjusting the dose or the formulation. This can improve the accuracy of diagnosis and the effectiveness of treatments, leading to better patient outcomes. This mechanism has developed as a main pathway to provide patients access to novel radiopharmaceuticals over recent years, especially in Europe (Hendrikse et al. 2022).

- Compassionate use

Another area where in-house compounding may address an unmet clinical need is in the area of compassionate use of radiopharmaceuticals that are still en route to MA. Compassionate use, also known as expanded access or pre-approval access, is a pathway that allows patients with severe or life-threatening conditions to access investigational drugs, including radiopharmaceuticals, outside of clinical trials when no other diagnostic or treatment options are available. The purpose of compassionate use for radiopharmaceuticals is to provide access to potentially beneficial treatments for patients who have exhausted all other options and are not eligible for clinical trials. This pathway requires sufficient evidence that the investigational radiopharmaceutical may be effective to treat or manage the condition, and there are no satisfactory alternative treatments.

The use of radiopharmaceuticals in compassionate use programs is typically guided by a treatment protocol that outlines the dosing, administration and monitoring procedures. The healthcare provider must follow the protocol and report any deviations to the producer and regulatory authorities. The main challenging requirement of this pathway is that the radiopharmaceutical has to be in active evaluation in clinical trials towards MA, which, for many radiopharmaceuticals with rare indications, is not the case. Additionally, the proprietary radiopharmaceutical producers may not always be willing to provide the radiopharmaceutical for compassionate use due to fears that it may negatively impact their clinical trial safety and efficacy data. In-house preparation of such radiopharmaceuticals for compassionate use addresses these challenges.

- Addressing MA-approved drug shortages

As has been recently witnessed by the nuclear medicine communities in certain regions of the world, the availability of the radiopharmaceuticals with MA in a certain region does not necessarily mean that the authorised producer will be able to continuously supply the drug to patients. Disruptions in radionuclide supply, logistical challenges and compliance with regulatory and quality requirements may all lead to temporary drug shortages from the commercial manufacturers. In those instances, the in-house producers should be allowed to produce those radiopharmaceuticals for the duration of the drug shortage in order to deliver these often life-saving medicines to patients. Similar consideration should also be given when addressing cases of very low or infrequent demand for MA-approved RPs faced in some countries. This necessitates adoption of the in-house preparation option of such RPs (subject to availability of an appropriate production facility and other resources), when deemed necessary for patients by the responsible physician.

### Foreseen regulatory challenges associated with new therapeutic radiopharmaceuticals (e.g. alpha emitters): availability and cost related to regulatory submissions

The development of new therapeutic approaches to human disease with radiopharmaceuticals has principally been focused in oncology and where recent pivotal clinical trials have demonstrated the safety and efficacy of these treatments (e.g. [^177^Lu]Lu-DOTATATE in patients with neuroendocrine tumours (NET) and [^177^Lu]Lu-PSMA ligand in metastatic castration resistant prostate cancer patients) and (relatively faster) inclusion in clinical practice guidelines (Bodei et al. 2022). The availability of these new radiopharmaceutical therapies is quite variable in different countries, and regulatory approvals have been achieved in only a small number of countries to date. This highlights an important issue of access to and availability of these new therapies and the regulatory processes for approval, when influenced by the small number of patients eligible for treatment (e.g. NET patients), formal industry regulatory submissions may not be forthcoming in most countries. In addition, the relatively high cost of formal regulatory submissions may be a barrier to allowing access to these treatments. To ensure that such patients can access these vital radiopharmaceutical therapies, a regulatory approach which facilitates access of patients to treatment in centres that have the laboratories and clinical facilities appropriate for production and treatment with such radiopharmaceuticals should be incorporated in the regulatory framework provisions.

The clinical development of radiopharmaceutical therapies which incorporate radionuclides that may not be currently used in licenced radiopharmaceuticals (e.g. copper-67, actinium-225, bismuth-213, lead-212, astatine-211) also warrants due consideration. The regulatory approaches should consider various aspects specific to each of such emerging radionuclides, keeping focus on the unmet clinical need of patients for these treatments. Certain regulatory challenges can be faced for actinium-225 labelled radiopharmaceuticals due to the complex decay chain of this radionuclide as well as due to co-production of the long-lived actinium-227 in some of the proposed production routes. It would be helpful to implement a pragmatic regulatory approach in such cases to support effective implementation of these innovative therapies. In the absence of such guidance, benefits of emerging radiopharmaceutical therapies to eligible cancer patients may be hampered.

## Summary and conclusion

There is a growing demand for utilisation of proven RPs for established and emerging clinical applications all over the world, including in many mid-to-low-income countries. Furthermore, recent developments in radiopharmaceuticals promise the emergence of clinically significant products for diagnosis of different disorders and screening, characterisation and targeted radionuclide therapy for certain cancers. The development of theranostic approaches using paired radiopharmaceuticals has principally been focused on in oncology and pivotal clinical trials have demonstrated the safety and efficacy of such treatments for cancer patients. There are however many associated challenges for translating such promising developments from bench to clinic and their wider deployment. Among the many challenges faced, obtaining timely regulatory approvals is a key requirement. Current pharmaceutical regulation practices in different countries have variable considerations for the distinct characteristics of RPs; in turn, different approaches are followed towards translation and use of new RP products, and many countries request guidance in this regard. This position paper is an outcome of a TM organised by the IAEA comprising a number of experts in the field to find a common understanding about the main considerations for RP regulations. These main considerations are:

- Various distinct characteristics of RPs and quality considerations based on their complexity (and nature)
- Requirements and mechanisms for licensing RPs, taking into account the limited number of preparations required for certain applications and the need to facilitate filing requirements to assure access to such RPs
- Means for ensuring access by domestic or regional supply
- Communication and a functional interface in regulatory bodies involved in RP regulations, including ways to ensure appropriate qualifications
- Specific regulatory requirements for the personnel involved in production, including those responsible for product release
- Appropriate quality assurance systems including GMP to meet the regulatory requirements

All these considerations for an appropriate regulatory environment for RPs must be reflected in the existing or the developing pharmaceutical regulatory framework in each country. It is essential to always consider that the main purpose of the regulations is to provide patients ease of access to “state-of-the-art” RPs with appropriate quality, safety and efficacy.

The TM strongly advocated creating an international expert group to provide regulatory guidance on RPs for IAEA MS. The IAEA is encouraged to establish, together with the WHO, such an expert group. Such a group could facilitate for many countries a common understanding of the requirements and compliance and serve to limit potential hurdles or challenges due to disparate understanding and expectations among the stakeholders.

## Data Availability

Not applicable.

## Abbreviations

EANM: European Association of Nuclear Medicine
FDA: (US) Food and Drug Administration
GMP: Good Manufacturing Practice
IAEA: International Atomic Energy Agency
IMP: Investigational medicinal product
IMPD: Investigational medicinal product dossier
MA: Marketing authorisation
MS: (IAEA) Member state
OECD: Organisation for Economic Co-operation and Development
PET: Positron Emission Tomography
PSMA: Prostate Specific Membrane Antigen
RP: Radiopharmaceutical
SmPC: Summary of Product Characteristics
SPECT: Single Photon Emission Computed Tomography
TM: (IAEA) Technical Meeting
WHO: World Health Organization
